# Genomic islands mediate environmental adaptation and the spread of antibiotic resistance in multiresistant *Enterococci* - evidence from genomic sequences

**DOI:** 10.1186/s12866-021-02114-4

**Published:** 2021-02-19

**Authors:** Weiwei Li, Ailan Wang

**Affiliations:** School of Life Science, |Ludong University, Yantai, 264025 China

**Keywords:** Genomic islands, *Enterococcus*, ARGs, Genome plasticity

## Abstract

**Background:**

Genomic islands (GIs) play an important role in the chromosome diversity of *Enterococcus*. In the current study, we aimed to investigate the spread of GIs between *Enterococcus* strains and their correlation with antibiotic resistance genes (ARGs). Bitsliced Genomic Signature Indexes (BIGSI) were used to screen the NCBI Sequence Read Archive (SRA) for multiple resistant *Enterococcus*. A total of 37 pairs of raw reads were screened from 457,000 whole-genome sequences (WGS) in the SRA database, which come from 37 *Enterococci* distributed in eight countries. These raw reads were assembled for the prediction and analysis of GIs, ARGs, plasmids and prophages.

**Results:**

The results showed that GIs were universal in *Enterococcus,* with an average of 3.2 GIs in each strain. Network analysis showed that frequent genetic information exchanges mediated by GIs occurred between *Enterococcus* strains. Seven antibiotic-resistant genomic islands (ARGIs) were found to carry one to three ARGs, *mdtG*, *tetM*, *dfrG*, *lnuG*, *and fexA,* in six strains. These ARGIs were involved in the spread of antibiotic resistance in 45.9% of the 37 strains, although there was no significant positive correlation between the frequency of GI exchanges and the number of ARGs each strain harboured (r = 0. 287, *p* = 0.085). After comprehensively analysing the genome data, we found that partial GIs were associated with multiple mobile genetic elements (transposons, integrons, prophages and plasmids) and had potential natural transformation characteristics.

**Conclusions:**

All of these results based on genomic sequencing suggest that GIs might mediate the acquisition of some ARGs and might be involved in the high genome plasticity of *Enterococcus* through transformation, transduction and conjugation, thus providing a fitness advantage for *Enterococcus* hosts under complex environmental factors.

**Supplementary Information:**

The online version contains supplementary material available at 10.1186/s12866-021-02114-4.

## Introduction

The genus *Enterococcus* is a gram-positive pathogen and is considered to be a leading cause of hospital-acquired infections [1, 2]. The rapid spread of enterococcal infection is due to the emergence of drug-resistant strains. The first vancomycin-resistant *Enterococcus* (VRE) appeared in Europe in 1986 [3], after which the increasing multidrug resistant VRE has brought new challenges to clinical treatment [4–7]. The high genome plasticity of *Enterococcus* makes it easy to acquire resistance through mutation and horizontal gene transfer (HGT) [2]. Genomic islands (GIs) are one of the important vectors for the acquisition of drug resistance.

GIs are clusters of genes within a bacterial genome with a specific GC% content and dinucleotide frequency and were first described by Hacker et al. [8]. GIs have diverse biological functions, such as pathogenicity, degradation of phenols, antibiotic resistance, iron uptake and secretory activity [9]. They also play an important role in genome plasticity, evolution and environmental adaption [8, 10, 11]. GIs are typically large pieces of DNA, ranging in size from a few kb to 500 kb, in which GIs below 10 kb are termed genomic islets [12]. An interesting feature of GIs is their transferability between organisms [10]. The transfer mode varies, mainly including conjugation, transduction and transformation [13–15]. GIs that are transferred by conjugation mode are also known as integrative conjugative elements (ICEs), which are one of the most widely studied types [16–20]. Type II and IV secretion systems are often associated with natural transformations of GIs. The SaPI GIs of *Staphylococcus aureus* and SGI1 GIs of *Salmonella* are transferred by transduction [21, 22]. Therefore, determining the GIs, especially the resistant GIs, is key for understanding the mechanisms of resistance gene transmission in multidrug resistant isolates.

With the development of whole genome sequencing, it has been gradually found that GIs are an important reason for the differences in microbial genomes [11, 18, 23–28]. Whether the high genome plasticity of *Enterococcus,* especially multiresistant *Enterococcus,* is closely related to GIs, is a topic worth studying. In view of this, some *E. faecium* and *E. faecalis* that carry multiple resistance genes were screened to investigate GI transmission and their correlation with antibiotic resistance genes (ARGs). The investigation of GIs and ARGs needs to be conducted based on the genome sequences of *Enterococcus*. However, only 179 sequences of the two species were assembled in the NCBI database, accounting for only approximately 5% of all sequencing strains. Fortunately, Sequence Read Archive (SRA) databases can provide abundant raw data that just need to be assembled before analysis. Here, the SRA data in NCBI and ENA (before December 2017) were explored with the help of the BIGSI web tool [29] to increase the representativeness and reliability of the data.

## Results

### Results of MLST

Of the 37 selected strains, 32 *E. faecium* belonged to 12 sequence types (STs), of which 26 were associated with hospital outbreaks of clade A (including CC-17 complex, e.g., ST18, ST20, ST22, ST56, ST80, ST117, ST192, ST203), six were nonhospitalized clade B (e.g., ST214, ST640, ST787, ST1246) [30–35], and five *E. faecalis* belonged to four STs, including hospital-associated infections (such as ST9 and ST40) and nonhospital-associated types (such as ST4 and ST64) [32] (Table s1). The diversity of STs indicated the complexity of the sample source, which made our investigation more reliable.

### GIs are extensively distributed in *Enterococcus*

A total of 119 GIs were found in 37 strains based on the prediction of IslandPath, with an average of 3.2 in each strain (Table s2). Strain 088817 contained the largest number of GIs with six, while strains 879,537 and 639,818 contained only one GI. The size of all GIs ranged from 2.045 kb to 33.622 kb, in which the smallest GI was present in strains 830,390 and 830,467, and the largest GI was present in strain 088817. We found 72 GIs < 10 kb (known as genomic islets), accounting for 60.5% of the total, and 47 GIs > 10 kb, accounting for 39.5% of the total, indicating that genomic islets are predominant in *Enterococcus* (Table s2). These results revealed that GIs were universal in *Enterococcus*.

### Prediction and correlation analysis of ARGs

**Fig. 1:**
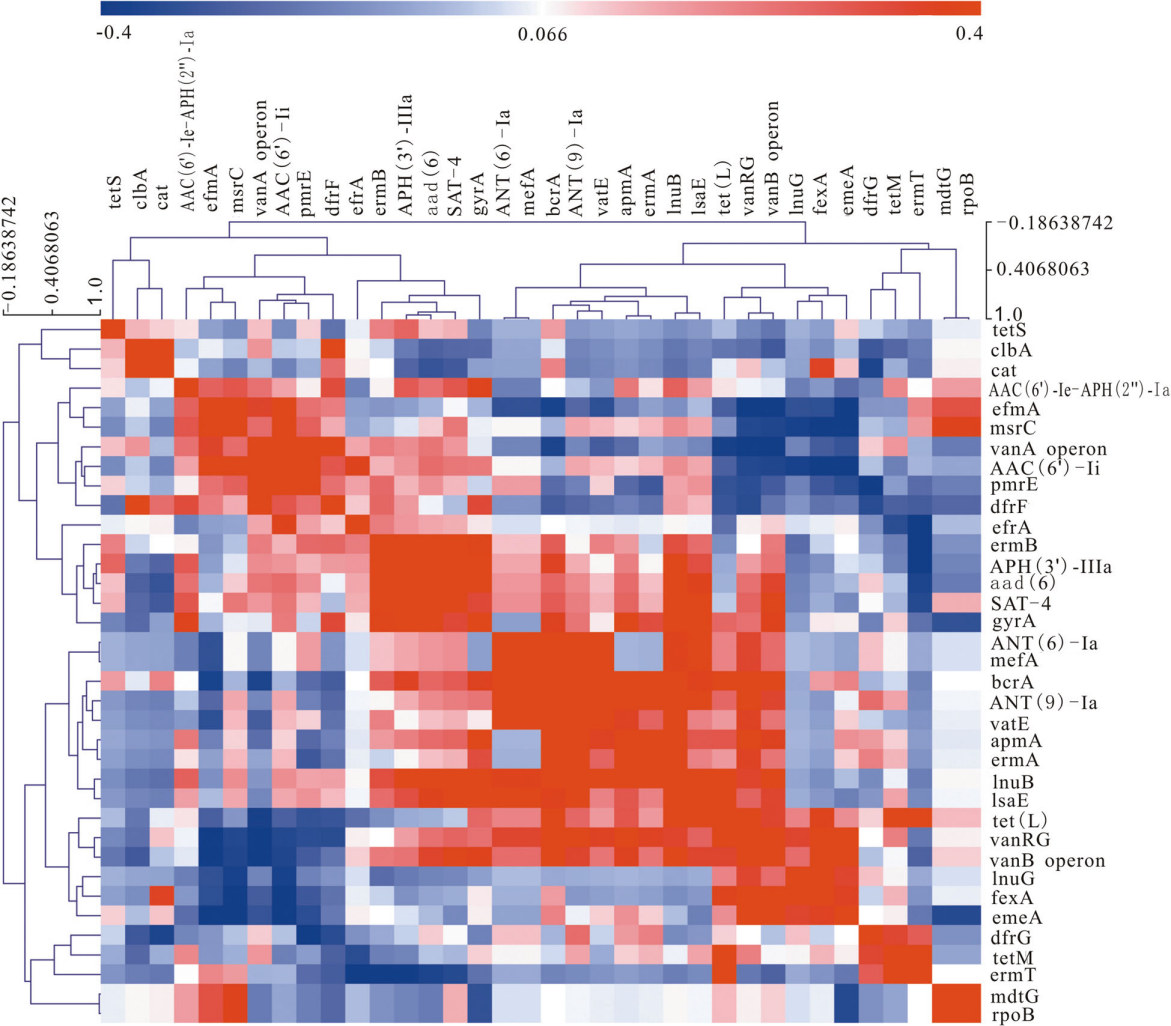
The correlation heatmap of ARGs. Blue represents low and negative correlations, and red represents high and positive correlations

The prediction results based on RGI software showed that all strains in this study were multidrug-resistant and carried multiple types of ARGs, including aminoglycosides (93%), diaminopyrimidines (100%), macrolides (84%), tetracyclines (79%), chloramphenicols (23%), lincoamides (16%), glycopeptides/peptides (100%), streptomycin (7%), streptothricins (40%) and multiresistant efflux pumps (100%) (Table s1, Fig. 1). The correlation heatmap of ARGs (Fig. 1) based on the Spearman correlation coefficient also showed that there were significant positive or negative correlations between ARGs (Table s3). For example, vanA operon was linked to *AAC (6′)-Ii*, *tet(L)*, dfrF, *PmrE*, *efmA*, *bcrA*, *vanB* operon, *vanRG*; *vanB operon* was linked to *aad (6)*, *ANT(9)-Ia*, *AAC(6′)-Ii*, *apmA*, *gyrA*, *tet(L)*, *SAT-4*, *msrC*, *lsaE*, *lnuB*, *vanRG*, *efmA*, *emeA*, *bcrA*, *fexA*; *vanG* Operon was linked to *AAC(6′)-Ii*, *ANT(6)-Ia*, *ErmA*, *dfrF*, *lnuB*, *lnuG*, *PmrE*, *vatE*, *msrC*, *mefA*, *ANT(9)-Ia*, *apmA*, *tet(L)*, *efmA*, *emeA*, *bcrA*, *fexA*, etc. There are many reasons for this phenomenon, the most likely of which is the possibility of colocalization or cotransfer of these genes.

### Discovery of antibiotic-resistant genomic islands (ARGIs)

**Fig. 2:**
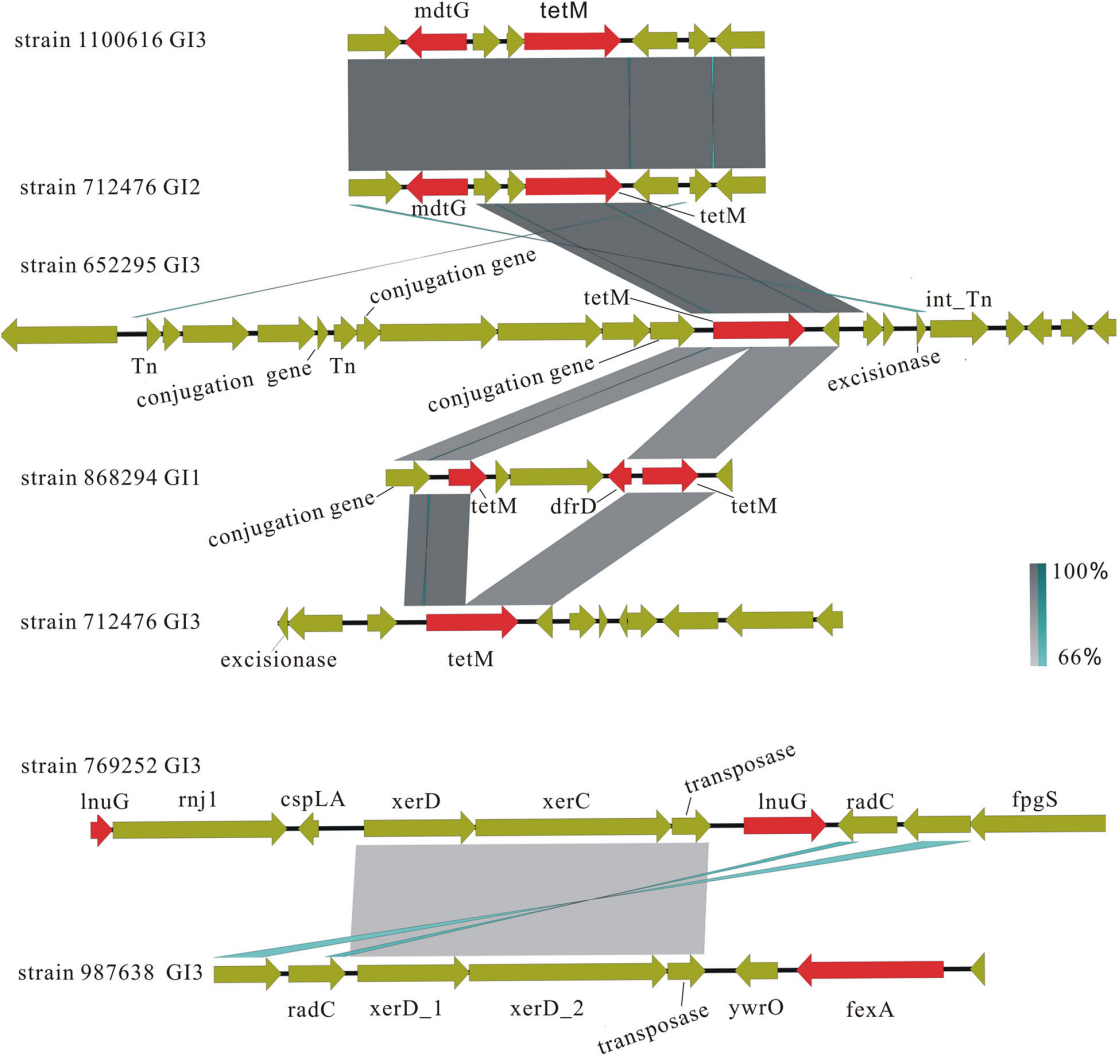
Linear comparison figures of ARGIs. The red arrows represent ARGs, the light green arrows represent other genes, and the blue connecting lines represent inversion. Unrelated genes are not labelled

By combining the prediction data of ARGs with GIs, we found seven GIs carrying ARGs in six *Enterococcus* strains, including strains 1,100,616, 712,476, 652,295, 868,294, 987,638, 769,252 (Fig. 2). As shown in Fig. 2, strain 712,476 contained two ARGIs, one (GI2) carrying *mdtG* (an efflux pump associated with fosfomycin resistance) and *tetM* (associated with tetracycline resistance) and the other (GI3) carrying *tetM*. Strain 1,100,616 GI3 and strain 712,476 GI2 contained the same ARG *mdtG*, and strain 1,100,616 GI3, strain 652,295 GI3 and strain 712,476 GI3 also contained the same ARG *tetM*. Strain 868,294 GI1 possessed 3 ARGs: *tet*M1, *tet*M2 and *dfrG* (diaminopyrimidine antibiotic resistance), and the first two genes had 86 and 90% amino acid identity with *tetM,* respectively, which was speculated to be its variant. Strain 769,252 GI3 carried one *lnuG* (lincosamide resistance) and one truncated *lnu*G. Strain 987,638 GI3 carried *fexA*, an efflux pump associated with chloramphenicol resistance. Further analysis showed that in addition to strains 1,100,616 GI3 and 712,476 GI2, the other five GIs all contained one or more mobility-related elements, such as conjugation genes, transposase genes or excisionase genes, suggesting their potential ability to carry ARGs for horizontal transfer. Interestingly, strains 1,100,616 GI3 and 712,476 GI2 had 100% similarity, and partial similarity was observed between strains 712,476 GI2, 652,295 GI3, 868,294 GI1 and 712,476 GI3. Partial similarity was also observed between strains 987,638 GI3 and 769,252 GI3, but no similarity was found between them and the above five GIs. These results suggested that some GIs in *Enterococcus* might mediate the spread of ARGs.

### Cluster analysis of GIs in *Enterococcus*

**Fig. 3:**
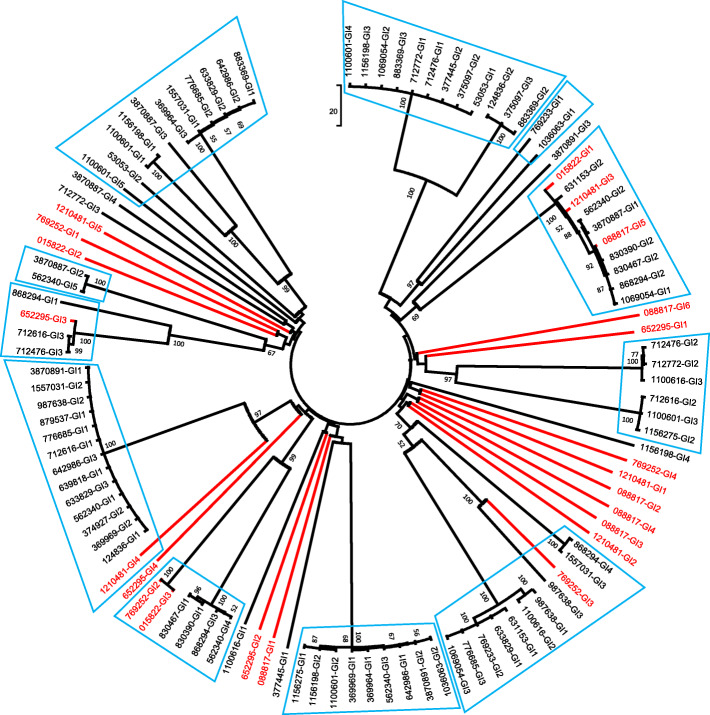
Cluster tree of GIs in *Enterococcus*. Bootstrap values (BS) greater than 50% are shown at the nodes. Eighty-seven GI sequences were clustered into 11 branches (BS ≥ 70%), and each branch is highlighted with a light blue box. The GIs of *E. faecalis* are shown in red, and the GIs of *E. faecium* are shown in black

To explore the spread of GIs in *Enterococcus*, a cluster tree was constructed by the neighbour-joining method based on 119 GI sequences using mega 7. After deleting the sequences that did not have any similarity to other sequences, we finally obtained a cluster tree based on 104 GI sequences (Fig. 3), and bootstrap values of ≥50% are shown at corresponding nodes. The deleted sequences were as follows: 015822 GI4, 015822 GI5, 530,353 GI2, 369,964 GI2, 374,927 GI1, 375,097 GI1, 375,097 GI4, 631,153 GI1, 642,986 GI2, 642,986 GI3, 769,233 GI3, 830,390 GI3, 830,467 GI3, 1,069,054 GI4, 3,870,887 GI2. It is generally believed that branches with bootstrap value (BS) ≥ 70% represent credible relationships [36], which suggests an exchange of genetic information between strains. As shown in Fig. 3, 87 GI sequences distributed in all 37 strains were clustered into 11 branches (BS ≥ 70%) (noted with a light blue box). Five GIs (GI1-GI5) of strain 562,340 were clustered in five different branches, four GIs (GI1-GI4) in strain 1,100,601 were clustered in four different branches, and three GIs (GI1-GI3) of strain 633,829 were clustered in three different branches, as did strains 776,685, 1,156,198 and 1,557,031. Some GIs of *E. faecalis* had also been found to be clustered with GIs of *E. faecium*, such as 015822 GI1, 088817 GI5, 652,295 GI3, 769,252 GI3 and 1,210,481 GI3. More results are not listed and can be found in Fig. 3. These results indicated that frequent genetic information exchanges mediated by GIs may occur within and between *Enterococcus* species.

### Transmission network of GIs in *Enterococcus*

**Fig. 4:**
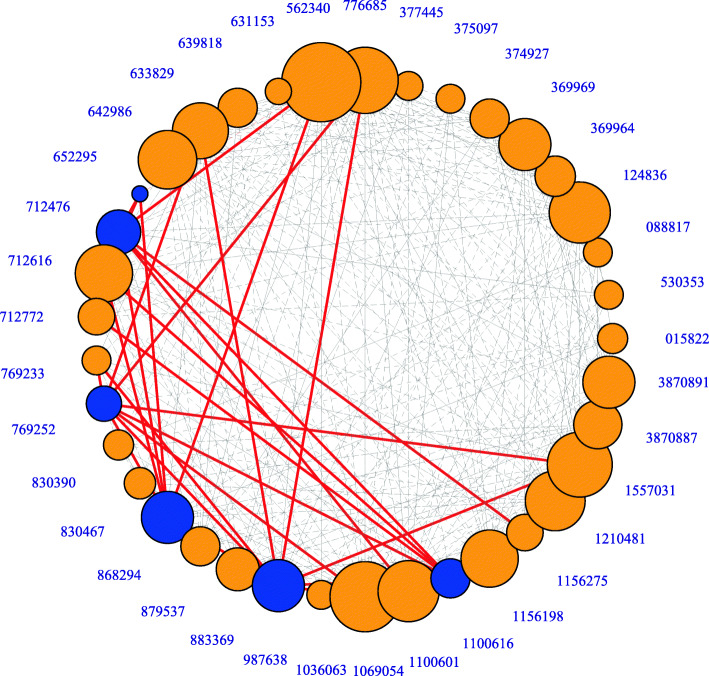
Network of GIs exchanges. The size of each circle is proportional to the frequency of GI exchange, the blue circles represent ARGIs and the orange circles represent non-ARGIs. Each grey line represents a GI exchange between the strain and another strain, and each red line represents a GI exchange mediated by ARGIs

To show the frequency and path of these exchanges more clearly, a network graph was built based on the above cluster analysis using R studio software (Fig. 4). In this network, the size of each circle was proportional to the frequency of GI exchanges. Strain 562,340 presented the most GI exchanges with other strains (27 times), and strains 1,069,054, 776,885, 1,557,031, 1,100,601, 1,210,481, 124,836, 1,156,198, and 712,616 also presented multiple GI exchanges (all > 20 times), while strain 652,295 presented just three exchanges with other strains. Although there were only seven ARGIs (harboured by six strains, the blue circles), their exchanges occurred in 17 strains (shown by red lines), accounting for 45.9% of the 37 strains. The analysis based on Spearman’s correlation coefficient (r = 0. 287, p<0.1) showed that there was no significant positive correlation between the frequency of GI exchanges and the number of ARGs each strain harboured (Table s4). These results suggest that ARGIs play an important role in the spread of some ARGs.

## Discussion

GIs are considered to be important tools of bacterial HGT and evolution [10]; therefore, they have become a research hotspot [11, 18–20, 28]. However, due to the variability of GI structure, the identification of GIs must be based on whole genome sequences (WGS). In the early days, the high cost of sequencing hindered the investigation of GIs. Tettelin et al. (2005) analysed the WGS of eight strains of *Streptococcus agalactiae* and found that each strain contained an average of 8 to 9 GI sequences [37]. Zhang et al. (2011) found several different GIs in *Klebsiella pneumonia* [38]. In the genus *Enterococcus*, several GIs have been reported to mediate strains to acquire new functions, such as virulence factors, vancomycin resistance or metabolic functions [18, 39–43]. In the current study, we investigated GIs in *Enterococcus* harbouring multiple ARGs and analysed their association with the transmission of ARGs. The SRA database provided sufficient sample data, and BIGSI helped us achieve an ultrafast search for the target strains. Our findings indicated that GIs are prevalent in *Enterococcus*.

Since GIs played an important role in the chromosome diversity of *Enterococcus*, we tried to trace their diffusion between strains by constructing a genetic information exchange network. Surprisingly, frequent exchanges of genetic information mediated by GIs were observed between all strains, including *E. faecium* and *E. faecalis*. In the cluster tree (Fig. 3), GIs from different species in *Enterococcus* were clustered together. Congruently, in the transmission network (Fig. 4), a total of 21 strains had 10–19 GI exchanges, and 11 strains had more than 20 GI exchanges, such as strain 562,340, which exchanged genetic information with 27 strains, and strains 776,685 and 1,069,054, which exchanged genetic information with 25 strains. These results indicated that the chromosomal diversity in *Enterococcus* might be related to the exchange of genetic information between different strains mediated by GIs. The frequent acquisition or loss of genetic information might be an important reason for the high genome plasticity of *Enterococcus*, which is of great importance for their adaptive evolution. From a Darwinian point of view, the driving force of evolution originated from environmental selection pressure [44]. The multidrug-resistant *Enterococcus* in this study was usually in a complex ecological niche, such as hospitals, care facilities, farms, water, stools, humans and pigs (Table s1), which was full of various adverse environmental factors, including antibiotic pressure, fluctuating temperature, and heavy metals. The GIs can be transferred, and in this process, they can carry multiple genes and integrate them into the bacterial chromosome, thus giving strain new metabolic functions to enhance its adaptability.

In this study, ARGIs were concerned. Of the seven ARGIs that we found, five contained mobility-related genes. For example, strain 652,295 GI3 contained three conjugation genes, three transposase genes, and one excisionase gene; strain 868,294 GI1 carried a conjugation gene; strain 712,476 GI3 contained an excisionase gene; and strains 769,252 GI3 and 987,638 GI3 each carried one transposase gene and two site-specific recombination enzyme genes (*xerC*, *xerD*). Both strains 769,252 and 652,295 belonged to *E. faecalis*, and their GIs exhibited some homology with that of *E. faecium*, suggesting that the spread of the ARGIs occurs intra- and inter-species (Fig. 2). In general, mobile GIs include some mobile elements, such as conjugative transposons and integrase genes [28, 45, 46]. It has been speculated that the mobility of GIs may originally originate from plasmids or bacteriophages. We predicted the plasmids and prophage sequences in all the strains based on contigs and compared them with the 119 GI sequences in this study. The results revealed that 27 GI sequences of 20 strains were adjacent or overlapped with those of prophages, and nine GI sequences of nine strains were carried by plasmids (Table s2), which partly supported the above hypothesis. However, this scan does not interpret the spread of all ARGs and GIs. We speculated that there may be other mechanisms that mediate the spread of ARGs and GIs, such as natural transformation.

We also found that strains 712,476 GI2 and 1,100,616 GI3 lacked the characteristics of typical ICEs (transposase, integrase, relaxase genes). They had 100% homology and carried the same ARGs *mdtG* and *tetM* (Fig. 2), suggesting that they originated from the same ancestor. The prophage and plasmid prediction results based on Phispy software showed that the two GI sequences were not located on the prophage or plasmid sequences (Table s2). Although the two strains belonged to different STs (ST203 and ST1246) and environments (hospital and farm), the lack of mobile vectors did not hinder the exchange of GIs. The two ARGIs are likely to be transferred through natural transformation. Natural transformation is one of the major mechanisms for HGT, in parallel with conjugation. The natural transformation of GIs depends on many factors, including the degree of homology between GI sequences and hosts, metabolic compatibility, environmental factors and endonuclease systems [10]. In gram-positive bacteria, naked DNA enters the cell via type IV pseudopili and can be integrated into the recipient’s genome by RecA-dependent homologous recombination [47]. It has been reported that some gram-positive bacteria (i.e., *Streptococcus pneumoniae*) are more likely to develop competence under antibiotic pressure [48], which may provide an advantage for the natural transformation of GIs. The species within the same genus *Enterococcus* also provided favourable conditions for homologous recombination between exogenous GIs and host chromosomes in vivo. We speculated that natural transformation might be the primary mode of GI transfer in *Enterococcus*, but this needs further study. The above results suggested that these GIs may contribute to the accumulation of ARGs in *Enterococcus* by multiple modes (conjugation, transformation, transduction). The correlation between the number of ARGs and the frequency of GI exchanges was also explored. The results showed that there was no significant positive correlation between them (Spearman’s correlation coefficient, r = 0. 287, *p* = 0.085). This is consistent with our investigation: only seven ARGIs were found, and although they mediated the exchanges of genetic information between nearly half of the 37 strains (45.9%) (Fig. 4), they were associated with the transmission of only five ARGs (Fig. 2). This indicates that GIs play a crucial role in the transmission of some ARGs but not all ARGs. In addition to GIs, other mobile genetic elements are also important carriers for the transmission of ARGs, such as plasmids and prophages. In addition, we cannot rule out the possible deviation due to the limited sample size in this study, and more data analysis is necessary.

The correlation between ARGs in *Enterococcus* was analysed with Spearman’s correlation test (Table s3, Fig. 1). One unanticipated finding was that multiple ARGs revealed significant (*p* < 0.05) or extremely significant (*p* < 0.01) correlations. Taking the vancomycin family genes as an example, the *vanA* operon had a significant positive correlation with four ARGs, the *vanB* operon had a significant positive correlation with 12 ARGs, and the *vanG* operon had a significant positive correlation with 12 ARGs. With this in mind, we analysed the possibility of colocalization of these ARGs and found that these genes were distributed in different contigs without colocalization. These strong correlations might be related to the coselection of environmental factors. It has been reported that heavy metals, biocides and antibiotics have coselection potential for bacterial ARGs [49]. The *Enterococcus* strains in this study originated from complex environments such as hospitals, farms, water, stool and care facilities (Table s1). Complex environmental factors might be potential coselectors to influence strain resistance, which might be a consequence of the selection of survivors in harsh environments. In addition, the strong correlations between ARGs may also represent the preference of *Enterococcus* for some ARGs in a complex environment, and this phenomenon deserves further investigation.

Finally, two limitations of this study were that 1) the source of strains used in this study only included multidrug-resistant bacteria in eight countries and seven kinds of environments, which could not represent all the regionally and environmentally resistant strains, and 2) the three ARGs (*cfr(B)*, *optrA*, *poxtA*) we retrieved did not appear in the prediction results of ARGs based on contigs. This may be due to the relatively low k-mer threshold (65%) chosen in the BIGSI web tool because it could obtain a larger range of raw reads. Nevertheless, these limitations did not affect the current analysis.

## Conclusions

The diversity and transferability of GIs may be an important factor for the chromosome plasticity of *Enterococcus*, which provides a fitness advantage for *Enterococcus* hosts under complex environmental factors. The data collected herein showed that the three modes of transformation, conjugation and phage-mediated transduction might exert an important role simultaneously in the GI transfer of *Enterococcus*. Another interesting result is that the distribution of many ARGs in *Enterococcus* showed a strong positive correlation, and whether this is relevant to coselection or cotransfer in complex environmental factors needs further study. Taken together, the current study provided some evidence based on genomic sequences about the distribution and spread of GIs in multiresistant *Enterococcus* and their effect on the accumulation of ARGs and metabolic functions.

## Materials and methods

### Screening of resistant strains

Bitsliced Genomic Signature Indexes (BIGSI) can retrieve 457,000 whole genome sequence (WGS) datasets submitted to SRA prior to December 2017 [25, 29]. A total of 17 ARGs were retrieved using the BIGSI web tool (available online: http://www.bigsi.io/). These ARGs are the most common genes conferring resistance to eight classes of antibiotics, including penicillin (*TEM*, *CTX-M*), glycopeptides (*vanA*, *vanB*), macrolides (*ermB*, *mphE*), tetracyclines (*tetM*, *tet( x4)*), quinolones (*qnrA*, *qnrD*), rifamycins (*arr-3*), chloramphenicols (*catI*, *floR*), oxazolidinones (*cfr(B)*, *optrA*, *poxtA*), and efflux pumps related to macrolide and quinolone resistance (*efmA*). In the end, a total of 156 results were obtained for eight ARGs (*vanA*, *vanB*, *cfr(B)*, *optrA*, *poxtA*, *efmA*, *ermB* and *TEM*) and none for the remaining nine ARGs (*CTX-M, mphE, tetM*, *tet(x4), qnrA*, *qnrD, arr-3, catI* and *floR*). We analysed and screened the metadata corresponding to 156 results and selected 37 sequencing data as representatives for subsequent investigation. The strains used for the 37 sequencing datasets were from eight different countries, including the United Kingdom, the United States, Germany, China, Australia, the Netherlands, Denmark and Japan, and were collected from different environments, such as hospitals, care facilities, farms, water, stools, humans and pigs (Table s1). All the selected strains were multidrug-resistant strains (resistant to three or more antimicrobial classes [50]), each containing 15–37 ARGs, with an average of 25.

### Bioinformatics

All genomic datasets were downloaded from ENA, and the raw reads were assembled into contigs using Shovill 1.0.9 (Available online: https://github.com/tseemann/shovill) by default settings. Genome annotation was performed using Prokka 1.13 with the UniProt database as the reference database [51] and NCBI BLASTn. ARGs were predicted using RGI 5.1.0 software [52] (evalue ≤1.0E-30, identity ≥50%) provided by the Comprehensive Antibiotic Resistance Database (CARD). IslandPath-DIMOB v1.0.4 [53] was combined with a script written by us (https://github.com/lwwal78/gbksplit) for GI prediction of multicontig data. Clustering analysis of GIs was performed using the neighbour-joining method by mega 7 software [54] with 1000 bootstrap replications. Packages “igraph”, “ggplot2”, and “scales” in R studio software were used to map the GI propagation network. Spearman’s correlation coefficient of ARGs was calculated using SPSS19, and the heatmap based on the correlation coefficient was generated by Mev 4.9. The maps of ARGIs were created by Easyfig 2.2.2 [55]. Multilocus sequence typing (MLST) was performed based on assembled contigs using MLST2.0 [56] provided by the Center for Genomic Epidemiology. Prophage sequences were predicted using PhiSpy 3.7.8 software [57]. Plasmid sequences were predicted using Miplasmids [58] (for *E. faecium,* https://sarredondo.shinyapps.io/mlplasmids/) and Plasmidfinder [59] (for *E. faecalis,* https://cge.cbs.dtu.dk/services/PlasmidFinder/).

## Supplementary Information

**Additional file 1: Table S1.** Sample information and ARGs in *Enterococcus; The same background color represents the resistance genes corresponding to the same class of antibiotics*.

**Additional file 2: Table S2.** GIs and phages in *Enterococcu*s; The yellow background represents GIs and prophages located in the same contig; The blue fonts represents GIs and plasmids located in the same contig.

**Additional file 3: Table S3.** Spearman’s correlation of ARGs in Enterococcus; ** represents p < 0.01, * represents P < 0.05.

**Additional file 4: Table S4.** Spearman’s correlation between ARGs and GI exchange frequency.

## Data Availability

All the genomic datasets can be retrieved from NCBI SRA or ENA, including the following SRA Accession numbers: *E. faecalis*: DRR015822, SRR088817, SRR652295, SRR1210481, ERR769252; *E. faecium*: ERR1036063, ERR1069054, ERR1100601, ERR1100616, ERR1156198, ERR1156275, ERR124836, ERR1557031, ERR369964, ERR369969, ERR374927, ERR375097, ERR377445, ERR562340, ERR712476, ERR712616, ERR712772, ERR769233, ERR776685, ERR830390, ERR830467, ERR868294, ERR879537, ERR883369, ERR987638, SRR3870887, SRR3870891, SRR530353, SRR631153,, SRR633829, SRR639818, SRR642986. In the analysis of this paper, we used the Arabic numerals in the Accession numbers as the name of the strains. To present clearer figures and results, we used the No. of strains in the full text instead of the name of strains, and their corresponding relationships can be found in Table S1.

## Abbreviations

GIs: genomic islands
ARGs: antibiotic resistance genes
BIGSI: Bitsliced Genomic Signature Indexes
SRA: Sequence Read Archive
WGS: whole genome sequences
ARGIs: antibiotic resistance genomic islands
VRE: vancomycin-resistant *Enterococcus*
HGT: horizontal gene transfer
ICE: integrative conjugative element
BS: bootstrap value
MLST: multilocus sequence typing
ST: sequence type
CC: clonal complex
